# Effect of Remote Essential Newborn Care Training Paired With In‐Person Quality Improvement on Optimizing Immediate and Resuscitative Care Practices

**DOI:** 10.1111/apa.70607

**Published:** 2026-05-26

**Authors:** Ashish KC, Prajwal Paudel, Pratiksha Bhattarai, Omkar Basnet, Rejina Gurung, Lea Kreyenbaum, Honey Malla, Beena D. Kamath Rayne, Sara Berkelhamer

**Affiliations:** ^1^ School of Public Health and Community Medicine University of Gothenburg Gothenburg Sweden; ^2^ Department of Pediatrics Paropakar Maternity and Women's Hospital Kathmandu Nepal; ^3^ Research Division Golden Community Patan Nepal; ^4^ Department of Women's and Children's Health Uppsala University Uppsala Sweden; ^5^ American Academy of Pediatrics Columbus Ohio USA; ^6^ Department of Pediatrics University of Washington Seattle Washington USA

**Keywords:** essential newborn care, health care provider's performance, in‐person quality improvement process, Nepal, remote training

## Abstract

**Aim:**

This study evaluated the effect of combining remote training (RT) with quality improvement (QI) processes on immediate and resuscitative care practices in a hospital in Nepal.

**Methods:**

This prospective, before‐after‐design study included 5034 newborns, with 1836 newborns independently observed for immediate and resuscitation care practice. Essential Newborn Care (ENC) training was delivered remotely via zoom to healthcare providers (HCPs) followed by six bi‐monthly facilitated QI interventions through Plan‐Do‐Study‐Act (PDSA) meeting. Generalized linear models were used to estimate the multivariable adjusted risk ratios (aRRs) between the periods as well as incidence risk ratio (IRR) change for key HCP performance on a weekly basis during PDSA interventions.

**Results:**

The proportion of deliveries for which resuscitation equipment was prepared by HCP increased from 9.6% to 43.7% between the observation periods (aRR = 1.44). Initiation of ventilation within the first minutes of birth improved significantly (0% vs. 18%, aRR = 1.49) with IRR increase by 17% with each additional week (IRR = 1.17; 95% CI; 1.08, 1.26). The proportion of infants who received repeat suctioning decreased non‐significantly (88.6% vs. 63.2%, aRR = 1.22).

**Conclusion:**

Pairing RT with facilitated QI led to improvements in immediate newborn and resuscitation care practice, recommending pairing of remote training and facilitating QI for future resuscitation program.

## Summary

**Why the Study Was Needed:**

- Neonatal resuscitation training can improve healthcare providers (HCP) performance but will require a quality improvement (QI) program to optimize its effect.

**What It Found:**

- Innovative pairing of ENC remote training (RT) with an in‐person facilitated QI program resulted in significant improvement in immediate newborn and resuscitative care practice.

**Implication for Clinical Practice:**

- Pairing of ENC remote training (RT) with facilitated QI can lead to improvements in HCP performance.

**Abbreviations**
BMV: Bag mask ventilation
ENC1: Essential Newborn Care Course, part 1
ENC Now!: Essential Newborn Care Now!
HBB: Helping Babies Breathe
HCP: health care provider
LMIC: Low‐ and Middle‐Income Country
NICU: Neonatal Intensive Care Unit
PDSA: Plan‐Do‐Study‐Act
PMWH: Paropakar Maternity and Women's Hospital
QI: quality improvement
RT: remote training
RTQI: Remote Training Quality Improvement
SD: standard deviation

## 1 Background

Over the past 20 years, efforts at the global, national and sub‐national levels to prioritize neonatal health in policies and programs have resulted in a 50% reduction in neonatal mortality [1, 2, 3]. To achieve an improvement in healthcare providers' (HCP) resuscitation performance to reduce neonatal mortality, initiatives like the implementation of Helping Babies Breathe (HBB) newborn resuscitation training, Quality Improvement (QI) programming, daily skills practice and clinical debriefing have been introduced [4, 5, 6].

In Nepal, we previously reported that disruptions to resuscitation training and QI efforts during the pandemic were associated with rapid declines in care quality and increased neonatal mortality in public hospitals [7]. As in‐person training of HCP in neonatal care and QI processes, including facilitated Plan‐Do‐Study‐Act (PDSA) cycles, was disrupted within an overburdened health system, routine QI activities could not be sustained [8, 9, 10]. The pandemic exposed vulnerabilities in health systems that rely heavily on in‐person education and routine QI processes. During the pandemic, interruptions to neonatal resuscitation training and QI activities were widely reported due to staffing shortages, reallocation of resources and restrictions on in‐person gatherings [11, 12, 13, 14].

In response to pandemic disruptions 2020, the American Academy of Paediatrics and Laerdal Global Health created an online platform (Helping Mothers and Babies Survive‐hmbs.org) to deliver the pre‐release version Essential Newborn Care (ENC) Course. This course updated HBB 2nd Edition to align with WHO guidelines and was subsequently released as the WHO ENC Curriculum [15, 16]. As with HBB, ENC online enabled remote, skills‐based learning through instructional videos and facilitated demonstrations, while continuing to emphasize hands‐on practice when feasible, supported by experienced facilitators [17]. As the digital platform allowed immediate access to ENC during the pandemic, it was initially called ENC Now!, but was later renamed ENC online.

Evidence has shown that neonatal resuscitation training improves resuscitation care, and the addition of quality improvement education can optimize care practice [18, 19, 20, 21, 22]. However, evidence on remote neonatal resuscitation training and in‐person quality improvement education on care practice is yet to be established. In this study, we sought to evaluate the effect of combining remote training (RT) in neonatal resuscitation with In‐Person QI education. In this innovative program, HCP received In‐Person QI education integrated into ENC1 training. This approach included reviewing local performance metrics alongside the training content. Following this, facilitated QI meetings were held to continuously review institutional data for gaps and implement changes using a PDSA model.

## 2 Patient and Methods

### 2.1 Study Design

This study used a prospective‐before and prospective‐after design to evaluate changes in HCP performance by combining remote neonatal resuscitation training with QI education and programming. The study was conducted with prospective data collection from September 2021 to May 2022, with September 1 to December 10, 2021 as the baseline period and December 11 to May 21, 2022 as the post‐intervention period.

### 2.2 Study Setting

The study was conducted in the high‐risk labour unit at Paropakar Maternity and Women's Hospital (PMWH) in Kathmandu, Nepal. Prior to the pandemic, this unit handled 20 to 24 deliveries per day with 8 delivery beds and an average of 20 000 deliveries annually. The unit has two resuscitation areas with resuscitation tables with neonatal resuscitation equipment (suction and resuscitator). The unit has 30 nursing staff for providing care for labour, delivery and early neonatal care. There was no change in the infrastructure and human resources before and during the interventions. Annual delivery rates in the hospital declined to 15 000 during the pandemic (March 2020–March 2022) in parallel with reduced staffing in the birthing centre and a high turnover of staff affected by COVID‐19 [23].

## 3 Human Resources

### 3.1 Participants and Inclusion Criteria

The participants included 10 HCP (nurse‐midwives) who attended deliveries to the high‐risk labour unit. Both providers with prior HBB training and those who were HBB naive were included to ensure that all staff were trained. For delivery observations, women at 22 weeks gestation or greater admitted to the labour room with foetal heart tones present on admission were included and consented to participate. Women with antepartum stillbirth, absent foetal heart sounds at admission, caesarean delivery, antenatally detected foetal anomalies, or twin or higher order gestation were excluded. Following delivery, cases with birthweight < 500 g were further excluded.

### 3.2 Intervention

The implementation of the RT In‐Person QI intervention was planned in three phases (i) Assessment of the need for Remote Training Quality Improvement (RTQI), (ii) Remote Training (RT) on ENC Now! and (iii) In‐person PDSA facilitation. During these phases, researchers from Nepal, Sweden and the US were present.

#### 3.2.1 Assessment of the Need for RTQI

An online zoom meeting was conducted with the hospital leadership (paediatric and nurse‐midwife) team to orient them to the *ENC Now!* RT package. The team included experienced ENC1 facilitators and focused on orientation to the newly adapted training materials. The local leadership team planned for the number of HCPs to be trained and addressed the logistics (training halls, mannequins, computer access, etc.) in the hospital for RT.

#### 3.2.2 Remote Training (RT) on ENC

Ten HCPs participated in remote training. Experienced ENC1 facilitators provided online demonstration of resuscitation skills while local hospital leadership facilitated group practice of the remotely demonstrated skills. Hospital leaders also reviewed baseline metrics applicable to each section of the training and QI methodology was taught. To allow for less disruption in clinical care, the content of ENC1 was broken up and covered along with QI processes in three sequential sessions.

#### 3.2.3 In‐Person PDSA Facilitation (QI)

The hospital paediatric consultant (PP) and nurse facilitator (PB) conducted in‐person PDSA sessions, including review of key metrics collected on the hospital's dashboard, which are presented in Table 1 with detailed operational definitions. Using the weekly progress collected on the ENC dashboard, the facilitator conducted six PDSA meetings between February 2 and April 25, 2022, with the HCP from the labour room. During each meeting, an ENC indicator with no demonstrated improvement was identified and root cause analysis was done using the PDSA diary. The proposed solution to the root cause was identified and an action plan was developed that would be implemented prior to the next meeting. In the subsequent meeting, interval progress was assessed, and further actions were determined (Table S1).

**TABLE 1:** Detailed operational definition of each indicator.

Indicator	Definition
Preparation of resuscitation equipment before birth	Resuscitation equipment (bag and mask, suction device, cord clamp, scalpel) checked and laid out prior to delivery.
Welcoming of the mother	HCP introduces and greets mother.
Practicing infection prevention	Hand washing with soap and water or alcohol‐based rub prior to delivery.
Explaining resuscitation procedures to mother	HCP verbally explains care procedures to the mother before, during or after immediate newborn care.
Initiation of skin‐to‐skin contact	Infant placed on mother's bare chest within first few minutes after birth.
Early initiation of breastfeeding	Infant put to breast within 1 h of delivery.
Delayed cord clamping (DCC)	Umbilical cord clamped between 1 and 3 min.
BMV within 1 min of birth	Among infants identified as non‐breathing at 30 s, initiation of BMV by 60 s after birth (‘Golden Minute’).
Ventilation quality	BMV delivered at rate of 40–60 breaths per minute.
Repeated suctioning	Suctioning of the non‐breathing infants in between ventilations.
Repeated stimulation	Stimulation of the non‐breathing infants in between ventilations.

### 3.3 Sample Size and Sampling Technique

Using the cohort sampling method, all women who came to the delivery unit and met the inclusion criteria were eligible for the study. Women who consented to the study were enrolled. To detect a 10% change in early initiation of breastfeeding with 80% power (*ß*) and 95% significance level (*α*), 1470 mother–newborn dyads would need to be enrolled in the pre‐intervention period, and the same sample would be needed in the post‐intervention period. Early initiation of breastfeeding was chosen for the sample size estimation as it can be objectively measured and has a standardized definition provided by WHO.

## 4 Data Collection

### 4.1 Clinical Observation

Research nurses were trained in data collection for ENC using a standardized observation checklist and were not engaged in the care provided at delivery. Observations included 60 h weekly, including both day and night shifts.

### 4.2 Implementation Level Data

Data was collected via multiple‐choice questions and a skills checklist to assess health workers' knowledge and skills before and after training. The PDSA diary documented continuous QI processes, including the problem identified, solutions proposed and actions taken.

### 4.3 Outcome Level Data

Data on socio‐demographic data, clinical practices and outcomes during intrapartum care were collected from patient charts. Since the aim was not improved documentation, but rather care practices, we are confident that the observable improvements reflect the care.

### 4.4 Data Management and Analysis

Data manager (OB) assessed the quality and completeness of the data. A data cleaning and analysis strategy was developed to ensure the accuracy of the data. Descriptive statistics were used to summarize maternal, neonatal and provider‐related characteristics between the baseline and intervention periods, using chi‐squared tests and non‐parametric tests. We estimated the change in HCP's performance before and after the intervention, using generalized Linear Models (GLMs) with a Poisson family, log link method. Adjustment variables included maternal age and education, as well as preterm birth status. Because of collinearity, no adjustment for birthweight was done. We calculated the incidence rate ratio of the practice that changes on a weekly basis during the intervention period using the Poisson regression model. Marginal predictions were computed from the adjusted models to estimate the predicted probability of each indicator across PDSA meetings.

## 5 Ethical Considerations

Ethical approval was obtained from the Institutional Review Committee of PMWH (IRC‐62/164) and Swedish Ethical Authority (Dnr 2024‐03941‐01). Written consent was obtained from all mothers whose deliveries were observed. Data were collected using a tablet‐based application and stored on a secure server to maintain confidentiality and prevent data loss.

## 6 Results

### 6.1 Health Workers Profile

The mean age of the participants was 31.0 ± 5 years, with 7.1 ± 4.2 years of experience and all of them were female. The knowledge score before vs. directly after training was 18.6 ± 2.1 versus 19.11 ± 2.3 (*p* = 0.457). Seven of 10 participants had received prior training on neonatal resuscitation.

### 6.2 Maternal and Infant Profile

At baseline, 2807 mother–newborn pairs were enrolled compared to 2227 during the intervention period. The mean maternal age was slightly higher in the baseline period (25.6 ± 4.7 years) than in the intervention period (24.9 ± 4.5 years, *p* = 0.001). The proportion of mothers with higher education increased significantly from 27.5% at baseline to 52.3% during the intervention (*p* = 0.001). The prevalence of preterm birth was slightly but significantly lower during the intervention period (8.3% vs. 11%, *p* = 0.001) (Table 2).

**TABLE 2:** Baseline and indicator characteristics of mothers before and after the RTQI intervention.

	Baseline	Intervention	*p* ^a^
	*n* = 2807	*n* = 2227	
Maternal age (mean, SD)	25.6 (SD 4.7)	24.9 (SD 4.5)	0.001
Educational status, *n* (%)			
Low education	121 (72.5%)	796 (47.7%)	0.001
High education	46 (27.5%)	873 (52.3%)	
Ethnicity, *n* (%)			
Disadvantaged	1857 (66.2%)	1464 (65.7%)	0.756
Advantaged	950 (33.8%)	763 (34.26%)	
Admission complication, *n* (%)			
No	2432 (86.6%)	1940 (87.1%)	0.622
Yes	375 (13.4%)	287 (12.9%)	
Low birth weight (LBW), *n* (%)			
No	2437 (87.1%)	1886 (86.2%)	0.357
Yes	360 (12.9%)	301 (13.8%)	
Preterm birth, *n* (%)			
No	2405 (89%)	2003 (91.8%)	0.001
Yes	297 (11%)	180 (8.3%)	

Abbreviation: SD, standard deviation.

^a^ *p*‐value generated from chi^2^ test.

### 6.3 PDSA Meetings

Four out of six PDSA meetings discussed the issue of delay in time to initiation of ventilation in non‐breathing infants, which were frequently attributed to excessive and prolonged suctioning and suctioning prior to starting BMV. A separate but related issue—interruption of ventilation due to repeated suctioning once BMV had been initiated—was also noted in discussions, although this was tracked as a distinct indicator on the heat map. Each PDSA meeting identified the bottlenecks for implementation with redesign of the setup and mechanics for resuscitation. Three out of six PDSA meetings identified inadequate greeting the mother, preparation at birth, handwashing practices and maintaining the infant skin‐to‐skin with the mother (Table S1).

### 6.4 HCP Performance Before and After RTQI

**FIGURE 1:**
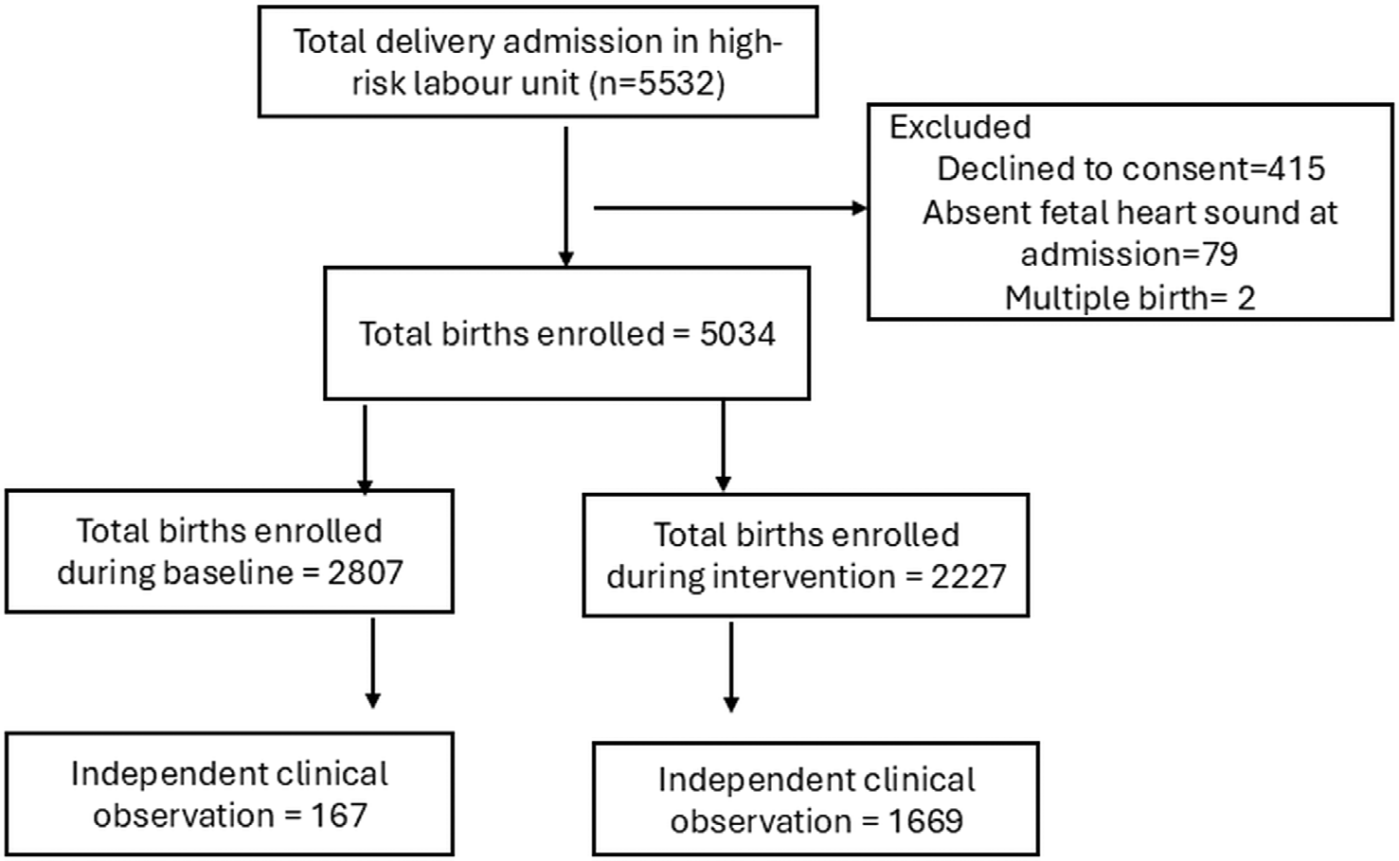
Study flow diagram during the study period.

Of the total (*n* = 2807) enrolled in the baseline period, 167 singleton newborns were observed and of the total (*n* = 2227) enrolled in the intervention period, 1669 were observed (Figure 1).

During the intervention period, significant improvements were observed in several key HCP's practices. Preparation of resuscitation equipment before delivery saw a substantial rise from 19.6% to 43.7% (aRR 1.44; 95% CI 1.40, 1.48). The practice of greeting mothers before delivery increased notably from 15.7% to 33.3% (aRR 1.29; 95% CI 1.24, 1.34), demonstrating an enhanced focus on patient interaction and comfort. In parallel, improved infection prevention before delivery has been observed with an increase from 16.8% to 36.7% (aRR 1.20; 95% CI 1.15–1.25). The practice of providing skin‐to‐skin contact for at least an hour after birth increased from 0.8% to 23% (aRR 1.10; 95% CI 1.09–1.11). Initiation of breastfeeding during the first hour after birth rose from 59.1% to 76.5% (aRR 1.04; 95% CI 1.03–1.06). The initiation of BMV within 1 min for non‐breathing infants increased from 0.0% during baseline to 22.0% during the intervention (aRR 1.49; 95% CI 1.18, 1.99). The optimal ventilation rate of 40–60 bpm decreased from 81.8% to 66.0% during intervention but was not statistically significant (aRR 1.09; 95% CI 0.97, 1.23). Repeated suctioning decreased from 58.3% during baseline to 23.5% during the intervention (*p* = 0.018). The analysis was adjusted for maternal age, education and preterm birth status (Table 3).

**TABLE 3:** Unadjusted and adjusted risk ratios for each indicator after RTQI intervention.

Indicator		Baseline (167)	Intervention (1669)	Unadjusted RR (95% CI)	Adjusted RR (95% CI)^a^
Preparation of resuscitation equipment, *n* (%)	No	151 (90.4%)	939 (56.3%)	Reference	Reference
	Yes	16 (9.6%)	730 (43.7%)	1.44 (1.40–1.48)	1.44 (1.40–1.48)
Welcoming of mother, *n* (%)	No	141 (84.4%)	1113 (66.7%)	Reference	Reference
	Yes	26 (15.6%)	556 (33.3%)	1.29 (1.24–1.34)	1.29 (1.24–1.34)
Practicing infection prevention, *n* (%)	No	139 (83.2%)	1057 (63.3%)	Reference	Reference
	Yes	28 (16.8%)	612 (36.7%)	1.20 (1.15–1.24)	1.20 (1.15–1.25)
Explanation to mother, *n* (%)	No	65 (38.9%)	386 (23.1%)	Reference	Reference
	Yes	102 (61.1%)	1283 (76.9%)	1.15 (1.09–1.13)	1.11 (1.09–1.13)
Initiation of skin‐to‐skin contact, *n* (%)	No	39 (24.5%)	246 (15%)	Reference	Reference
	Yes	120 (75.5%)	1390 (85%)	1.01 (0.99–1.02)	1.00 (0.99–1.02)
Duration of skin‐to‐skin contact, *n* (%)	No	119 (99.2%)	1070 (77%)	Reference	Reference
	Yes	1 (0.8%)	320 (23%)	1.1 (1.09–1.16)	1.1 (1.09–1.11)
Initiation of breastfeeding, *n* (%)	No	65 (40.9%)	383 (23.5%)	Reference	Reference
	Yes	94 (59.1%)	1247 (76.5%)	1.04 (1.03–1.06)	1.04 (1.03–1.06)
Cord Clamping between 1 and 3 min, *n* (%)	No	18 (11.3%)	183 (11.2%)	Reference	Reference
	Yes	141 (88.7%)	1453 (88.1%)	1.02 (1.00–1.03)	1.02 (1.01–1.03)
BMV within Golden Minute, *n* (%)	No	11 (100%)	39 (78%)	Reference	Reference
	Yes	0 (0%)	11 (22%)	1.35 (1.05–1.74)	1.49 (1.18–1.99)
Ventilation at 40–60 bpm, *n* (%)	No	2 (18.2%)	17 (34%)	Reference	Reference
	Yes	9 (81.8%)	33 (66%)	1.2 (1.07–1.36)	1.09 (0.97–1.23)
Repeated suctioning, *n* (%)	No	5 (41.7%)	39 (76.5%)	Reference	Reference
	Yes	7 (58.3%)	12 (23.5%)	1.06 (0.71–1.58)	1.22 (0.85–1.74)
Repeated stimulation, *n* (%)	No	28 (58.3%)	172 (70.5%)	Reference	Reference
	Yes	20 (41.7%)	72 (29.5%)	0.89 (0.75–1.04)	0.92 (0.79–1.08)

^a^ Adjusted for maternal age, education and preterm birth status.

### 6.5 HCP Performance on Weekly Basis After Initiation of In‐Person PDSA Meeting

Using a Poisson regression model, we assessed the incidence rate ratio (IRR) for health workers performance for each additional week after initiation of in‐person PDSA meeting. With each additional week during the intervention, there was 19.4% increase in the rate of preparation for resuscitation equipment than the previous week (IRR = 1.19; 95% CI; 1.17, 1.21, *p* < 0.001). The IRR for practice of infection prevention increased by 13% with each additional week (IRR = 1.13; 95% CI; 1.11, 1.14, *p* < 0.001). The IRR for immediate breastfeeding increased by 2% with each additional week during the intervention period (IRR = 1.02; 95% CI; 1.01, 1.03, *p* < 0.001). The IRR for initiation of bag and mask ventilation within first minute (golden minute) increased by 17% with each additional week (IRR = 1.17; 95% CI; 1.08, 1.26, *p* < 0.001). The IRR for repeated suctioning decreased non‐significantly by 5% with each additional week (IRR = 0.95; 0.86, 1.05, *p* = 0.348). The IRR for repeated stimulation decreased by 4% with each additional week (IRR = 0.96; 0.93, 0.99, *p* = 0.006) (Table 4).

**TABLE 4:** Incidence rate ratio per week of indicator after initiation of in‐person PDSA meetings.

Indicator	Incidence rate ratio (IRR) per week	95% CI	*p*
Preparation of resuscitation equipment	1.19	1.17, 1.21	< 0.001
Welcoming of mother	1.13	1.11, 1.14	< 0.001
Practicing infection prevention	1.08	1.07, 1.09	< 0.001
Explanation to mother	1.03	1.02, 1.04	< 0.001
Initiation of skin‐to‐skin contact	0.99	0.95, 1.00	0.003
Immediate breast feeding	1.02	1.01, 1.03	< 0.001
Cord clamping after 1–3 min	1.0	1.0, 1.0	0.353
BMV within Golden Minute	1.17	1.08, 1.26	< 0.001
Ventilation at 40–60 bpm	1.02	0.99, 1.02	0.181
Repeated suctioning	0.95	0.86, 1.05	0.348
Repeated stimulation	0.96	0.93, 0.99	0.006

*Note:* Predicted probabilities derived from marginal effects analysis demonstrated upward trends in most indicators over continuous PDSA meetings (Figure 2a–k).

## 7 Discussion

RTQI of ENC are associated with significant changes in routine care practices including marked improvement in rates of preparing resuscitation equipment, welcoming mothers, handwashing before delivery and maintaining skin‐to‐skin contact after delivery. Resuscitation practices improved, with significant change in the rates of DCC and BMV within the Golden Minute, while ventilation at an appropriate rate, repeated suctioning and stimulation did not change, despite being addressed in QI meetings, indicating the need for additional skill‐focused QI interventions. Marginal effects analyses showed mostly progressive increases in predicted probabilities across successive PDSA cycles, highlighting the cumulative benefit of repeated meetings.

**FIGURE 2:**
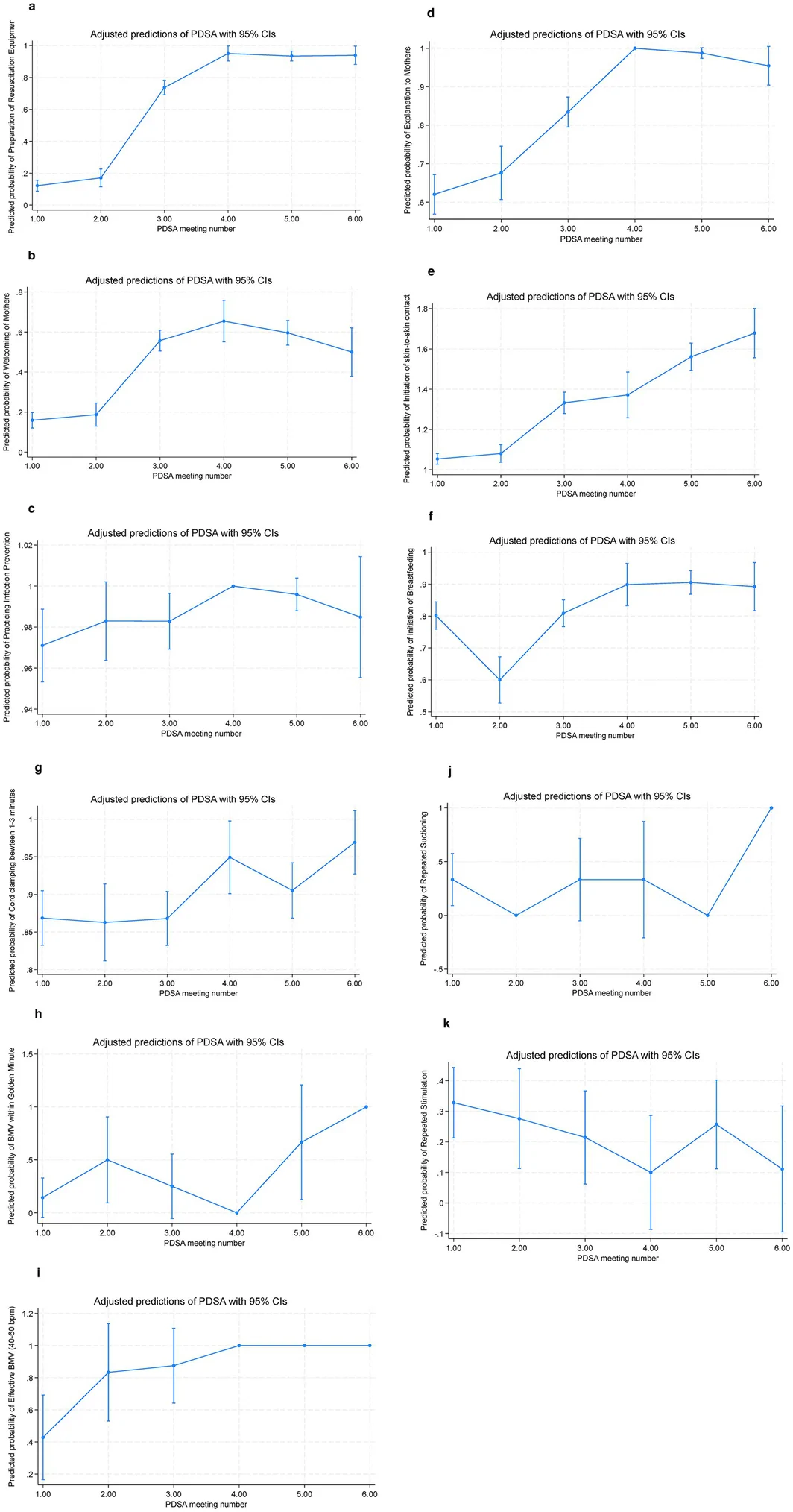
Predicted probability of (a) preparation of resuscitation equipment, (b) welcoming of mothers, (c) practicing infection prevention, (d) explanation to mothers, (e) initiation of skin‐to‐skin contact, (f) initiation of breastfeeding, (g) cord clamping between 1 and 3 min, (h) BMV within Golden Minute, (i) effective BMV (40–60 bpm), (j) repeated suctioning, and (k) repeated stimulation, by PDSA meeting.

The patterns of improvement in Table 4 and Figure 2a–k suggest that remote training and serial PDSA meetings gradually improve respectful care, handwashing before delivery, skin‐to‐skin contact of newborns and resuscitation practice. Each PDSA meeting identified bottlenecks for implementation to identify the barriers to progress so that overcoming them can change performance in subsequent PDSA meetings. For example, facilitators and HCPs identified that the reduction in stimulation of non‐breathing infants was key to reducing the time to first ventilation. Redesigning the delivery room to move the resuscitation table closer to the delivery bed also significantly changed the time to first ventilation. This suggests that PDSA was much more effective in improving complex skills and that further hands‐on practice would complement performance.

The PDSA meeting provided a participatory approach to identify the problems each HCP faced during immediate newborn and resuscitation care. The engagement of the multi‐disciplinary team (paediatric and nursing) to understand the bottlenecks of the problem facilitated setting a common goal for the next PDSA meeting. The continuous PDSA meeting to review the action plan implemented based on the set goals helped improve team performance and is reflected in the results. However, there were certain performances that still did not change despite the identification of performance problems such as repeated suctioning which interrupted bag and mask ventilation rates. These key performances might require more PDSA meetings over a period of time.

We note that welcoming mothers, explaining the procedure of care, preparing equipment, handwashing and practice of early skin‐to‐skin were discussed during five out of six PDSA meetings which also likely enhanced the impact of our intervention on these outcomes. The second wave of the COVID‐19 outbreak was occurring during the intervention and contributed to HCP's poor adherence to equipment preparation at the beginning of our study. In addition, gaps in knowledge around the safety of skin‐to‐skin care during the outbreak were addressed during the PDSA meeting. Currently, the American Academy of Paediatrics (AAP) is facilitating the translation of ENC training into practice through the rollout of the Customized MentoRship and Implementation Support Package (CRISP), including in humanitarian settings [24].

Our findings of improved provider practices with RTQI are consistent with broader evidence that structured training coupled with QI processes can enhance neonatal care delivery. Systematic reviews have shown that implementation of basic resuscitation training programs on HBB together with quality improvement intervention is associated with reductions in early neonatal mortality and intrapartum stillbirth rates in low‐resource settings [22, 25, 26]. Prospective evaluations of scaled QI intervention packages in similar contexts have demonstrated improvements in resuscitation performance and faster initiation of ventilation [8, 20, 27, 28]. Furthermore, multi‐component QI strategies that include regular skill checks and review meetings support retention of neonatal resuscitation skills over time [29]. Lastly, broader QI frameworks that combine PDSA cycles, data feedback and mentoring have also been effective in improving ENC practices across diverse settings, especially in combination with leadership engagement, reinforcing the potential value of our hybrid intervention [30, 31].

## 8 Limitations

We acknowledge multiple limitations to the study. First, despite planned 24‐h coverage, independent observation of all deliveries was not feasible, and approximately 25% of deliveries during the intervention period were not observed. Observations were dependent on data collector availability during periods of high clinical workload, which may introduce self‐selection bias. In line with this, differences in care practices by day versus night shifts were not assessed and may have influenced provider performance. The other limitation is the higher maternal education level observed during the intervention period may have contributed to improved care practices through increased patient engagement, representing a potential source of residual confounding. Moreover, this is an observational study design used to evaluate an intervention where the intervention is not actively assigned to control and intervention group, meaning that the RTQI interventions were introduced through a natural phenomenon. Concurrent evaluation of our intervention in a control and intervention group would be of interest to establish causal linkage but requires a multisite study. However, we used GLMs to control the temporal changes in performance and to assess the effect of serial PDSA processes on performance. Additionally, our study was performed in a large public referral hospital which may limit how applicable our findings are in other hospital settings and since we excluded women who had caesarean section and higher birth order, the results are not generalizable to all women. Finally, the observation was conducted by independent trained nurses, which might have introduced bias in the recording of clinical events and inaccuracies in the dataset. To reduce the impact of observation bias, a standard observation checklist was developed and reviewed tools on a bi‐weekly basis.

## 9 Conclusion

The results of RTQI implementation in a tertiary hospital of Nepal demonstrate that during restricted movement such as a pandemic or emergency, RT paired with in‐person PDSA can bring about change in many aspects of HCP performance. In‐person PDSA ensures a team approach to understand the problems and use a participatory approach to tackle the problem, which is a sustainable approach. However, challenges remain in terms of scalability of PDSA in rural areas as facilitation using the tool would require competency gain within a group of HCPs. Further to implement a zoom based remote training in remote will require a dedicated internet access, which is a challenge in rural settings of Nepal.

The implementation of ENC through Remote Training and In‐person PDSA can improve HCP performance for routine practices of newborn care. However, further implementation research is required on strategies to optimize and improve HCP performance on skills performed with neonatal resuscitation.

## Author Contributions

**Omkar Basnet:** conceptualization, methodology, software, supervision, project administration, visualization, writing – review and editing. **Pratiksha Bhattarai:** conceptualization, methodology, validation, supervision, project administration, writing – review and editing. **Ashish KC:** conceptualization, investigation, writing – original draft, funding acquisition, project administration, supervision. **Rejina Gurung:** conceptualization, methodology, data curation, supervision, project administration, resources, writing – review and editing. **Lea Kreyenbaum:** writing – original draft, formal analysis, visualization, investigation. **Honey Malla:** methodology, data curation, supervision, project administration, visualization, writing – review and editing. **Prajwal Paudel:** conceptualization, methodology, data curation, supervision, project administration, writing – review and editing. **Sara Berkelhamer:** conceptualization, methodology, data curation, resources, funding acquisition, writing – review and editing, validation, investigation. **Beena D. Kamath Rayne:** conceptualization, resources, supervision, visualization, writing – review and editing, validation, investigation.

## Funding

This work was supported by a grant from the Laerdal Foundation for Acute Medicine (2021) (S.B., A.K.C.). The funder had no role in the design and conduct of the study.

## Conflicts of Interest

The authors declare no conflicts of interest.

## Supporting information


**Table S1:** Content and agreements of PDSA meetings.

## Data Availability

The data that support the findings of this study are available from the corresponding author upon reasonable request.
