# Supplementary material for: Effect of Remote Essential Newborn Care Training Paired With In‐Person Quality Improvement on Optimizing Immediate and Resuscitative Care Practices
Source: Acta Paediatr. 2026 May 26;115(10):2106–15. doi: 10.1111/apa.70607 (PMC13552586; doi:10.1111/apa.70607)
Supplement: Supplementary file 1 [file APA-115-2106-s001.docx]

Table S 1. Content and Agreements of PDSA Meetings.

| **No. of PDSA Meeting** | **Date** | **No. of participants** | **Problems Identified** | **Problems Priortized** | **Root cause** | **Possible Solutions** | **Responsible person** | **Time Period** |
| --- | --- | --- | --- | --- | --- | --- | --- | --- |
| 1st | 2nd February, 2022 | 10 | 1. Unable to achieve golden 1 minute for resuscitation. 2. Lack of skin-to-skin contact continuation for 1 hour 3. Lack of handwashing in each delivery. 4. Lack of respectful maternity care. 5. Delay initiation of breastfeeding. 6. Delay cord cutting. 7. Lack of preparation of equipment (resuscitation). | Unable to achieve target of golden 1 minute for resuscitation | 1. Over stimulation 2. Over suctioning 3. Delay cord clamping 4. Lack of preparation of resuscitation equipment | 1. Decrease the stimulation time. 2. Prevent unnecessary suctioning. 3. Prompt action to non- breathing babies. 4. Prepare equipment prior delivery. | On duty staff  supported by incharge | 2 weeks |
| 2nd | 23rd February, 2022 | 14 | 1. Unable to achieve golden 1 minute for resuscitation. 2. Lack of skin-to-skin contact continuation for 1 hour 3. Lack of handwashing in each delivery. 4. Lack of respectful maternity care. 5. Lack of effective ventilation in babies receiving bag and mask / neopuff ventilation. | Unable to achieve target of  golden 1 minute. | 1. Over stimulation  2. Over suctioning  3. Distance between delivery table and resuscitation corner. | 1. Decrease stimulation  and suctioning.  2. Keep high risk women near resuscitation table | On duty staff  supported by incharge | 2 weeks |
|  |  |  |  | 2. Ineffective ventilation (40 - 60 bpm). | 1. Lack of proper skill. | 1. Practice skill drill.  2. Practice on advance neonatalie / upright bag and mask with golden community. |  |  |
| 3rd | 7th March, 2022 | 7 | 1. Lack of handwashing 2. Lack of greeting to mothers. 3. Lack of continuous skin-to-skin contact  4. Lack of babies receiving ventilation within golden 1 minute. 5. Lack of Breast-Feeding | 1. Lack of ventilation in Golden 1 minute. 2. Lack of continue skin to skin contact in crying babies. | 1. Lack of Manpower. 2. Lack of resuscitation preparation. 3. Delay in cord clamping in non-breathing babies.  1. Lack of counselling to mother. | 1. Early cord cutting in non-breathing babies. 2. Early preparation of resuscitation equipment. 1. counselling to mothers. | Duty staffs | 10 days |
| 4th | 4th April, 2022 | 10 | 1. Lack of Handwashing. 2. Unable to meet skin to skin contact for 1 hour among crying and breathing babies. 3. Unable to meet the target of Golden 1 Minute ventilation among non-breathing babies. 4. Unable to maintain RMC. 5. Articles preparation (lack of preparation for resuscitation equipment) 6. Unable to monitor FHS of 100% client. | 1. Lack of handwashing  and sanitization. | 1. Lack of awareness.  2. Lack of time (spontaneous delivery, wheelchair delivery).  3. Attitude (Individual attitude). | 1. Share the importance of handwashing amon the co-workers and students.  2. Change in attitude and behaviour. | On duty staff  supported by incharge | * |
|  |  |  |  | 2. Golden 1 minute ventilation among non- breathing babies. | 1. Distance between delivery bed and resuscitation corner. | 3. Keep the high-risk woman near resuscitation table. |  |  |
| 5th | 15th April | 10 | 1. Lack of handwashing  2. Lack of greeting mothers inside labour room.  3. Minimising timing of baby receiving 1st Ventilation  4. Not placing baby in skin-to-skin contact for 1 hour. | 1. Lack of handwashing before delivery | 1. Lack of attitude and practice. | 1. Change in practice and attitude. | On duty staff  supported by incharge | 1 week |
|  |  |  |  | 2. Not placing baby in skin- to skin contact. | 2. Lack of knowledge, attitude and practice. | 1. Changing practice, attitude and knowledge. Addressing and notifying time of skin-to skin contact and keeping baby skin to skin |  |  |
| 6th | 25th April | 11 | 1. Lack of handwashing before  conducting delivery. | 1. Lack of handwashing before  conducting delivery. | 1. Individual Attitude  towards the practices. | 1. Change in attitude  and do more practices.   2. To give orientation to all new health workers. | On duty staff  supported by incharge | 1 week |
| Note: * indicates no any comments from health worker. | | | | | | | | |
